# Proportions of the Human Figure

**Published:** 1892-02

**Authors:** 


### PROPORTIONS OF THE HUMAN FIGURE

Dr. Giovanni, of Milan, has recently published in Italian, a remarkable work on the *morfologia* of the human body, which promises to work quite a revolution in the study of disease. Among other interesting facts presented by this eminent investigator are the following respecting the proper proportions of the ideal human figure :

1. The height of a person is equal to the greatest stretch of the arms; that is, the distance between the tips of the middle fingers when extended laterally as far as possible.

2. The circumference of the chest is equal to one-half the height.

3. The length of the sternum, or breast bone, is equal to one-fifth of the circumference of the chest.

4. The height of the abdomen, measuring from the pubic bone to the end of the sternum, not including the ensiform cartilage, is two-fifths the circumference of the chest. The umbilicus marks the middle point between the pubic bone and the lower end of the sternum.

5. The greatest distance between the large bones of the pelvis is four-fifths the height of the abdomen.

Represented in inches these measurements are as follows for a man and a woman who closely approach the ideal type:

| | Man. | | Woman. | |
|---|---|---|---|---|
| Height | 68.8 | in. | 64 | in. |
| Extreme stretch of arms | 68.8 | " | 64 | " |
| Circumference of chest | 34.5 | " | 31.8 | " |
| Length of sternum | 6.8 | " | 6.4 | " |
| Height of abdomen | 12.9 | " | 12 | " |
| Width of pelvis | 10.4 | " | 10.1 | " |

